# A novel pathway for multiscale high-resolution time-resolved residual stress evaluation of laser-welded Eurofer97

**DOI:** 10.1126/sciadv.abl4592

**Published:** 2022-02-16

**Authors:** Bin Zhu, Yiqiang Wang, Jiří Dluhoš, Andy J. London, Michael Gorley, Mark J. Whiting, Tan Sui

**Affiliations:** 1 Department of Mechanical Engineering Sciences, University of Surrey, Guildford, Surrey GU2 7XH, UK.; 2 United Kingdom Atomic Energy Authority, Culham Centre for Fusion Energy, Culham Science Centre, Abingdon, Oxon OX14 3DB, UK.; 3 TESCAN ORSAY HOLDING, a.s., Libušina třída 21, 623 00 Brno, Czech Republic.

## Abstract

The plasma-facing components of future fusion reactors, where the Eurofer97 is the primary structural material, will be assembled by laser-welding techniques. The heterogeneous residual stress induced by welding can interact with the microstructure, resulting in a degradation of mechanical properties and a reduction in joint lifetime. Here, a Xe^+^ plasma focused ion beam with digital image correlation (PFIB-DIC) and nanoindentation is used to reveal the mechanistic connection between residual stress, microstructure, and microhardness. This study is the first to use the PFIB-DIC to evaluate the time-resolved multiscale residual stress at a length scale of tens of micrometers for laser-welded Eurofer97. A nonequilibrium microscale residual stress is observed, which contributes to the macroscale residual stress. The microhardness is similar for the fusion zone and heat-affected zone (HAZ), although the HAZ exhibits around ~30% tensile residual stress softening. The results provide insight into maintaining structural integrity for this critical engineering challenge.

## Teaser

Multiscale, high resolution and time-resolved residual stress is quantified in laser-welded Eurofer97 fusion reactor steel.

## INTRODUCTION

Nuclear fusion is a potential substitute source of electricity production to solve dependence on fossil fuels, reduce carbon emissions, and provide a major contribution to net zero targets. The in-vessel components in the fusion plant, such as pipes, breeding blanket, and divertor cassette, have to use complex materials systems, complicated joining techniques, and maintenance processes to enable their function under extreme operating conditions (*1*, *2*). Laser welding is a promising technique that is used extensively in a wide range of industries to overcome the intrinsic assembly and maintenance difficulties. Previous studies have demonstrated the feasibility of using remote laser tools to butt-weld in-vessel components (*3*, *4*).

Reduced-activation ferritic/martensitic (RAFM) steels, which are an evolution of high Cr Grade 91 steel, are widely used as structural materials in various in-vessel components for the DEMOnstration power plant (DEMO). Eurofer97, one of the RAFM steels, uses lower activation elements like tungsten, vanadium, and tantalum in appropriate quantities. It is used as the European reference material for the EU-DEMO reactor because of its excellent mechanical properties: creep life, fracture, strength, and ductility (*5*, *6*). When joining Eurofer97, laser welding does, however, induce significant residual stresses, up to c.800 MPa, as a result of the nonuniform deformation caused by the thermal cycle and the martensite phase transformation, which takes place after welding (*7*, *8*).

The residual stress largely originates from strain misfit between different regions and is usually categorized into three types according to the length scale (*9*). Type I (macroscale) residual stress is usually measured by averaging over a range of grains in a region ranging from micrometers to millimeters and varies continuously across the material. The microscale residual stress includes the type II residual stress, which arises from microstructural misfit, and the type III residual stress from the defects and dislocations induced by the welding process. Macroscale residual stress can affect the fracture of the material, while microscale residual stress aggravates cracking at grain level under in-service elevated temperature. Evaluating the multiscale residual stress and microstructure and their impact on the as-welded Eurofer97 is crucial to determining joint reliability and developing predictive tools for the in-vessel component of DEMO.

Many techniques can be used to characterize the residual stress distribution in metallic materials, e.g., neutron diffraction (*7*, *8*, *10*), hole drilling (*11*), and laboratory-based and synchrotron-based x-ray diffraction (XRD) techniques (*12*–*14*). For example, some attempts have been made to study the residual stress distribution in laser-welded Eurofer97 by the neutron diffraction, where the resolution is over a millimeter for macroscale characterization (*8*, *10*). Microscale residual stress characterization is therefore neglected because of low resolution. The XRD techniques achieve high-resolution (HR) characterization but sacrifice penetration. The limited penetration sometimes limits the sample dimensions, raising concerns of residual stress relief in thin and small samples, and it is hard to measure the residual stress precisely for highly textured material (*12*, *15*).

Digital image correlation (DIC) is one of the strain measurement techniques (*16*, *17*) that is capable of characterizing average strain over a region and HR strain maps by combining with other microscopy-based techniques. For example, HR electron backscatter diffraction (EBSD) and DIC method is an established technique for microscale residual strain characterization (*18*). However, requiring a stress-free reference is challenging, and the technique only characterizes type III residual strain. Ga^+^ focused ion beam (FIB) and DIC method has proven reliable in measuring the time-resolved strain relaxation in titanium alloys, metallic glasses, and martensitic steels (*19*–*21*). Although it measures residual strain without a stress-free reference, two notable limitations exist, which affect residual strain measurements: (i) the accelerated Ga^+^ ions damage the material by amorphizing, creating defects and increasing dislocation density, which is likely to induce residual stress during the Ga^+^ FIB milling process (*22*), and (ii) to achieve a multiscale residual stress characterization of metallic alloys, the low removal rates of Ga^+^ FIB usually limit the milling areas to a few micrometers, which largely achieves only type III residual stress (*20*).

The relatively new Xe^+^ plasma focused ion beam (PFIB) technique gives rise to less material damage and larger volume removal within a reasonable acquisition time (*23*). Thus, the Xe^+^ PFIB provides a potential solution for multiscale residual stress (types I, II, and III) characterization. Combining the Xe^+^ PFIB with the DIC to the laser-welded Eurofer97, macroscale residual stress can be directly measured by averaging over multiple grains within each gauge volume (i.e., milled pillar), ranging from millimeters to micrometers. Simultaneously, the microscale residual stress localization in the milled pillar can be visualized by time-resolved HR strain maps.

In this study, the Xe^+^ PFIB-DIC technique was used to evaluate the multiscale residual stress in laser-welded Eurofer97 steel. The macroscale residual stress was obtained, and the time-resolved HR strain map was used to visualize the microscale residual strain field, which provides insights into the initiation and propagation of creep cracking. Nanoindentation was also used to cross-validate the residual stress distribution from the Xe^+^ PFIB-DIC technique. The microhardness was then evaluated for the quantitative analysis of residual stress hardening and microstructural hardening. The mechanistic connection between residual stress, microstructure, and microhardness was established, which contributes to optimizing the manufacturing process and addressing the structural integrity problems raised by residual stress.

## RESULTS

### Microstructural characterization

**Fig. 1.**
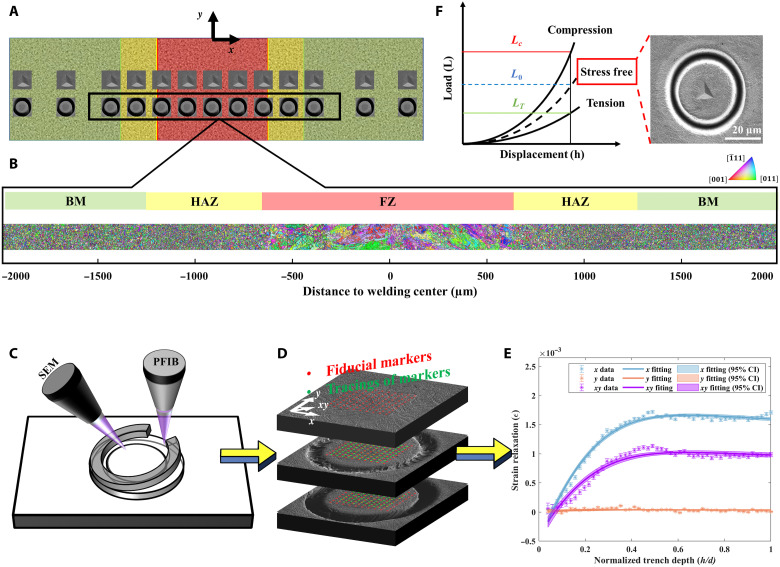
Experimental schematic illustration and microstructural characterization. (**A**) Schematic figure showing the areas and positions of EBSD mapping, nanoindentation, and PFIB-DIC. (**B**) Stitching map showing the grain size, shape, and orientation. (**C**) Schematic figure illustrating SEM image acquisition during Xe^+^ PFIB incremental milling steps. (**D**) Illustration of the tracking of the ring core, using the displacement between fiducial mesh and the mesh after milling increments. (**E**) Strain relaxation profiles at incremental normalized trench depth (*h/d*) and master fitting curve along the *x*, *y,* and *xy* directions, and the error bar shows the 95% CI. (**F**) Schematic figure of residual stress measurement by nanoindentation and an example of location-dependent stress-free reference.

The Materials and Methods provides material and experimental details of Eurofer97 steel, laser conditions of welding processing, and sample preparation of the laser-welded sample. The coordinates are defined in Fig. 1A, where the *x* direction is horizontal, and the laser welding direction (*y*) is vertical. The EBSD was used to characterize grain size and orientation distribution in the region marked by the black rectangle (4750 × 200 μm^2^) in Fig. 1A. The sample is divided into three regions, fusion zone (FZ), heat-affected zone (HAZ), and base material (BM), according to the peak temperature history of Eurofer97, where melting occurs in the FZ region, and austenitization and the martensite transformation occur in the HAZ region. The grain morphology covering the three regions is illustrated in Fig. 1B, made by stitching together 19 separate EBSD maps. The average grain size was extracted from EBSD orientation maps by the mean linear intercept method (*24*). The grains had an average size of 11 ± 2.10 μm in the FZ region, whereas in the HAZ and BM regions, the grains show the average size of 6 ± 0.92 and 7 ± 1.05 μm, respectively. The stitched EBSD map shows the microstructural transition area at the center of the FZ region and interfaces between FZ-HAZ and HAZ-BM regions. The grains in the FZ region with an obvious preferred orientation arise from the heat flow during the laser welding, which is the origin of the texture (*25*, *26*).

### Residual stress distribution characterized by Xe^+^ PFIB-DIC and nanoindentation method

The Xe^+^ PFIB-DIC technique was applied to evaluate the multiscale residual stress of laser-welded Eurofer97. The incremental milling step using the Xe^+^ PFIB releases residual stress gradually as the milling depth increased, and the DIC technique visualizes the strain relaxation and enables residual stress measurement. The ring-core method was applied as it allows simultaneous evaluation of three components of in-plane normal and shear strain relaxation (Δε*_x_*, Δε*_y_*,and Δε*_xy_*) (*27*). Figure 1C schematically shows the ring-core incremental milling steps and scanning electron microscopy (SEM) acquisition processes. The incremental milling was stopped when the depth was equal to the ring-core diameter to ensure that the residual stress is fully released in the ring-core region (*27*–*29*). The ring-core displacement that results from the ring-core expansion, or shrinkage, during incremental milling is recorded by the markers (the displacement between red and green markers in Fig. 1D by DIC). The strain of each marker is extracted from the gradient of the displacement. In addition, averaging of the strain of markers in the ring-core region enables measurement of the strain relaxation at each trench depth, and the “master curve” fitting evaluates the three components of the macroscale strain relaxation values (Fig. 1E) (*30*). The derivation of the strain relaxation in three orthogonal directions enables direct calculation of the principal strain relief using Mohr’s circle (*31*). Inversion of the sign of the perceived strain relaxation provided a means of determining the residual strain, and the residual stress was then calculated using Hooke’s law (*32*).

The nanoindentation measurements that also provide in-plane residual stress distribution were performed to cross-validate the results from the Xe^+^ PFIB-DIC ring-core method and establish the microstructural and residual stress hardening effect. As shown in Fig. 1F, the equi-biaxial residual stress was extracted by comparing the load and contact area at the same depth between residual stress and stress-free state (*33*). Performing indentation on the ring core where the residual stress is fully released after PFIB milling provides the location-dependent stress-free state and stress ratio. The location-dependent stress-free reference avoids differences arising from changes in microstructure during laser welding.

**Fig. 2.**
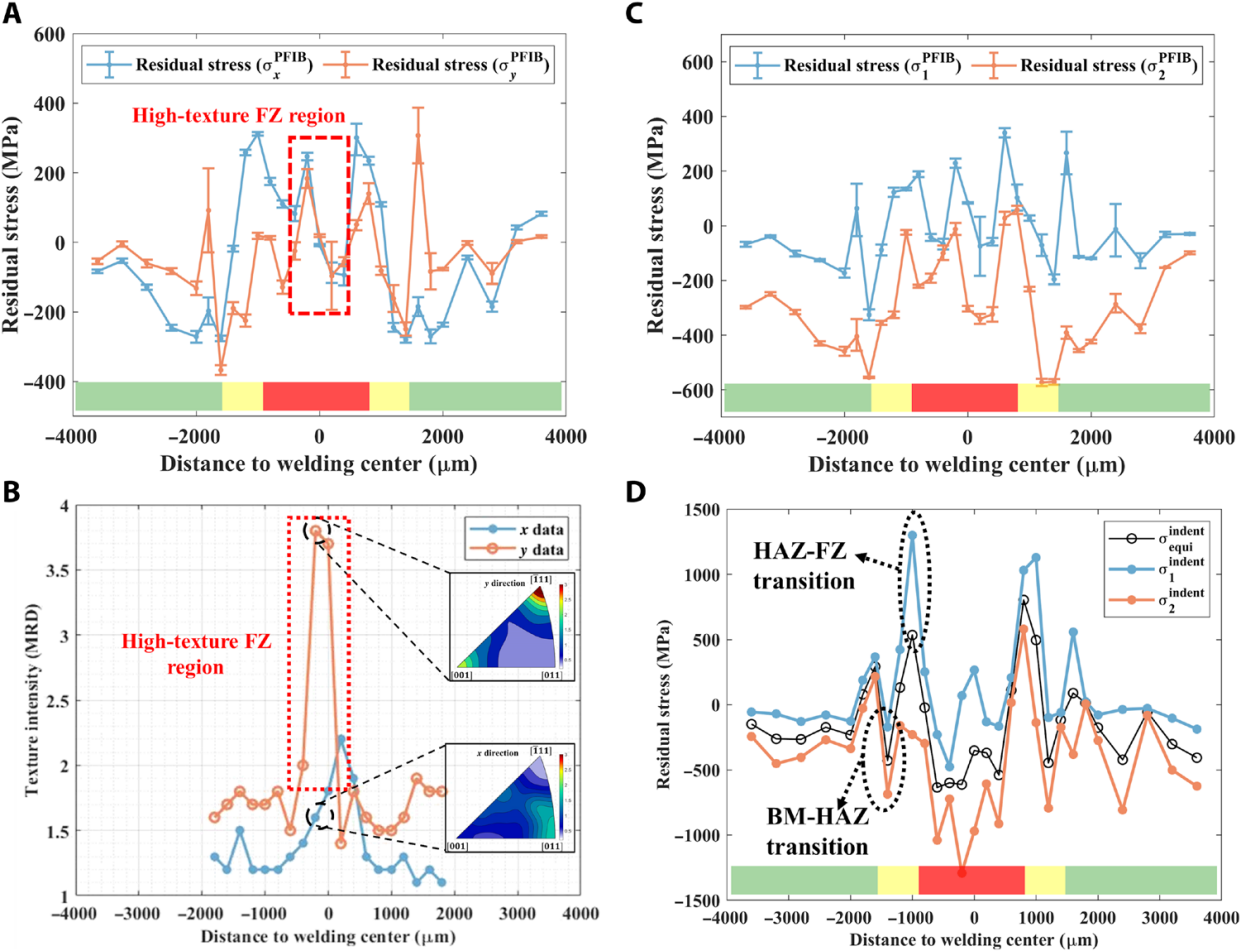
Residual stress distribution across the weldment. (**A**) Residual stress distribution in the *x* and *y* directions measured by Xe^+^ PFIB-DIC ring-core method. (**B**) Texture distribution across the weldment in the *x* and *y* directions, and the example IPF in the FZ region. (**C**) Principal residual stress distribution across the weldment from Xe^+^ PFIB-DIC ring-core method. (**D**) Equi-biaxial and non–equi-biaxial residual stress distribution measured by nanoindentation residual stress measurement.

Performing a line scan across the weldment in the EBSD mapped regions by the Xe^+^ PFIB-DIC ring-core method aims to establish the residual stress distribution and microstructural correlations in the FZ, HAZ, and BM regions. The line-scan resolution is 200 μm in the FZ and HAZ regions and 400 μm in the BM region, as limited residual stress in the BM region is expected because of the relatively low thermal input of laser welding. It can be seen in Fig. 2A that the peaks of tensile residual stress are observed at both the FZ-HAZ and HAZ-BM interfaces, balanced by the adjacent compressive residual stresses in the HAZ region. The texture intensity in multiples of random distribution (MRD) in the *x* and *y* directions is identified using inverse pole figures (IPFs). Analyzing the texture intensity of the 19 separate EBSD maps enables comparison of the texture across the weldment (Fig. 2B). An asymmetric residual stress profile is also identified in the high-texture FZ region, as shown in Fig. 2A. Figure 2C illustrates the distribution of two principal residual stress components, which also exhibit peak values at the interfaces. The principal residual stress profile is also used for cross-validating the PFIB-DIC ring-core method by comparing it with the nanoindentation residual stress measurements.

Figure 2D reveals that the residual stress distribution as measured by the nanoindentation method. The peak of compressive residual stress in the FZ is as large as −500 MPa, whereas the peak of tensile residual stress with the value up to 800 MPa occurs around the interface of FZ and HAZ regions. When moving away from the centerline of the weldment, the residual stress decreases to c. –400 MPa before reaching the BM region. Unlike the PFIB-DIC ring-core method, the nanoindentation technique has been proven to evaluate the residual stress with the assumption of equi-biaxial residual stress distribution (*33*). Compared with the residual stress distribution from the Xe^+^ PFIB-DIC ring-core method, the profile from nanoindentation measurements shows the symmetric residual stress distribution with higher magnitudes due to the assumption of an equi-biaxial stress state.

To cross-validate the Xe^+^ PFIB-DIC ring-core method, the equi-biaxial residual stress from the nanoindentation measurement is transformed to non–equi-biaxial residual stress by using location-dependent stress ratio (*k*) derived from the two principal residual stress components measured by the Xe^+^ PFIB-DIC technique. The detail of this transformation can be found in Materials and Methods. The non–equi-biaxial residual stress profile of nanoindentation measurements (Fig. 2D) displays a similar trend as the results of the Xe^+^ PFIB-DIC technique, although the peaks of tensile residual stress in the microstructural transition areas around the FZ-HAZ and HAZ-BM interfaces show an unexpected lack of agreement regarding magnitudes (compared with Fig. 2C). This apparent discrepancy may arise because of the selection of the stress-free reference and stress ratio. The peak value of residual stress occurs at the interface of FZ-HAZ and HAZ-BM, which is consistent with the results from the Xe^+^ PFIB-DIC ring-core method.

### Time-resolved HR multiscale strain relaxation analysis

**Fig. 3.**
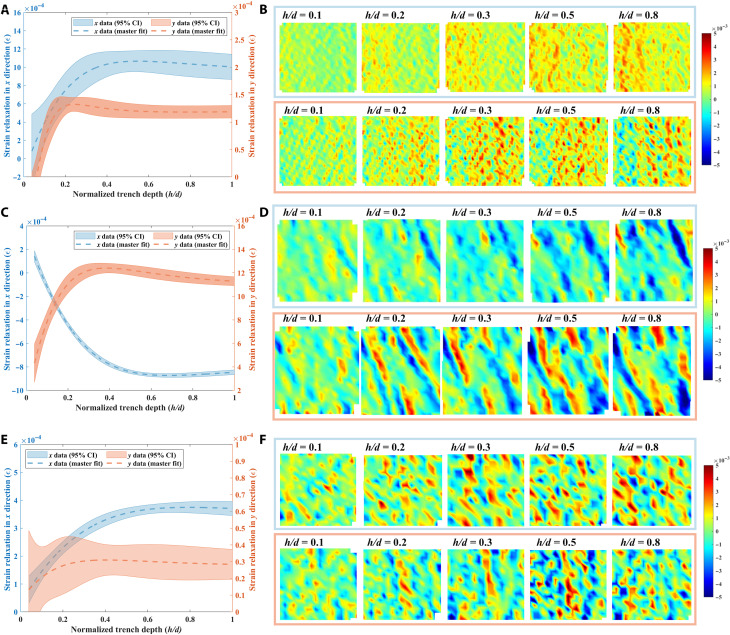
The time-resolved multiscale strain relaxation during Xe^+^ PFIB milling step. (**A**) Strain relaxation profile of the ring core at high-texture FZ region in the *x* and *y* directions with 95% CI. (**B**) Corresponding time-resolved HR strain relaxation maps in the *x* and *y* directions. (**C**) Strain relaxation profile of the ring core at low-texture HAZ region in the *x* and *y* directions with 95% CI. (**D**) Corresponding time-resolved HR strain relaxation maps in the *x* and *y* directions. (**E**) Strain relaxation profile of the ring core at low-texture HAZ region in the *x* and *y* directions with 95% CI. (**F**) Corresponding time-resolved HR strain relaxation maps in the *x* and *y* directions.

The time-resolved strain relaxation profiles and maps were evaluated quantitatively with example ring cores in high-texture FZ and low-texture HAZ and BM regions to explore the multiscale strain relaxation. The strain relaxation profiles (Fig. 3, A, C, and E) show the macroscale strain relaxation averaged over the ring core during the stress relief, whereas the HR strain maps (Fig. 3, B, D, and F) show microscale information at higher resolution in the ring core. Comparing the profiles and the maps demonstrates the importance of monitoring the microscale strain relaxation during residual stress relief. The strain relaxation profile (Fig. 3A) presents the macroscale strain relaxation of the ring core in the high-texture FZ region, which shows the higher strain relaxation values of 1.2 × 10^–3^ in the *x* direction than the value of 1.05 × 10^–4^ in the *y* direction. The localized microscale strain relaxation was identified by these HR maps (Fig. 3B), where significantly higher magnitude was found in the *y* than in the *x* direction in the ring core. The magnitude of localized microscale strain relaxation is higher than macroscale strain relaxation profile for both examples at high-texture FZ and low-texture HAZ. Significant microscale strain relaxation is also found in the BM regions, although the macroscale strain relaxation is much smaller than in the FZ and HAZ regions. It also shows that microscale tensile strain relaxation in the region was subjected to compressive strain relaxation macroscopically and vice versa. Furthermore, although the strain relaxation profiles in Fig. 3 (A and C) remain stable when the value of *h/d* reaches 0.4, the time-resolved HR strain relaxation map still captures the microscale strain. These findings indicate that using the time-resolved HR strain relaxation analysis enables evaluation of the microscale strain relaxation during the stress relief, which is neglected in the macroscale strain relaxation profile.

### HR microscale residual strain relaxation correlated with crystal microstructures

**Fig. 4.**
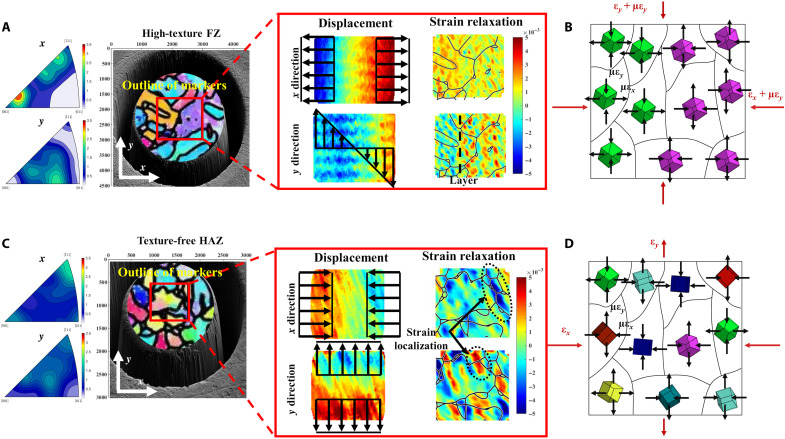
Examples of displacement and strain relaxation mapping in the high-texture FZ and low-texture HAZ regions. (**A** and **C**) The texture intensity and the crystallographic orientation of the ring cores and the outline of the fiducial marker array (red rectangle). The displacement maps of the ring core and the HR strain relaxation maps overlaid by the grain boundary in the *x* and *y* directions indicate the strain partitioning and strain relaxation localization. (**B** and **D**) Schematic figures explain the texture effects on residual stress evaluation by the PFIB-DIC ring-core method.

To explore and visualize the role of microscale residual stress quantitatively, the displacement and HR strain relaxation of the ring core were mapped and correlated with microstructure. Two ring-core measurements were selected from the high-texture FZ region and the low-texture HAZ region to demonstrate the displacement and HR strain relaxation maps. It can be seen in Fig. 4 (A and C) that the overlaid EBSD maps enable visualization of the crystallographic orientation, and the outline (red dashed rectangle) reveals the position of the markers. The displacement maps disclose the corresponding deformation of the ring cores because of the residual stress relief. Closer inspection of the displacement maps reveals a uniform distribution in the *x* direction for both ring cores and in the *y* direction for the ring core in the low-texture HAZ region, while a linear distribution is found in the high-texture FZ region in the *y* direction. The difference in texture intensity between these two regions is assumed to result in this linear distribution potentially. The IPF shows that the grains have a preferential orientation in the *x* and *y* directions for the pillar in the high-texture FZ region. This implies that the grains with different crystallographic texture show distinct deformation behavior during strain relief, resulting in the microscale residual stress not being in local equilibrium in the ring-core region.

By calculating the displacement gradient, the HR microscale strain relaxation was quantitatively evaluated and mapped. The distinct heterogeneous localized microscale strain relaxation was observed mostly around the grain boundaries in both high-texture FZ and low-texture HAZ regions. However, because of the difference regarding grain orientation in the pillar, a layer between tensile and compressive region is found in the FZ region, whereas no such layer is found in the low-texture HAZ region (Fig. 4C). In Fig. 4 (B and D) are the schematical figures showing the measured residual stress and localized texture effect. The cubes in the figure represent the grain in different orientations. In the high-texture region, most grains display similar microscale residual stress, resulting in nonequilibrium strain relaxation during the ring-core milling. In this case, the microscale residual stress significantly affects the residual stress distribution. In contrast, the microscale residual stress is self-equilibrated in the low-texture region, and the measured residual stress distribution is equivalent to the macroscale residual stress.

### Microstructural and residual stress hardening

**Fig. 5.**
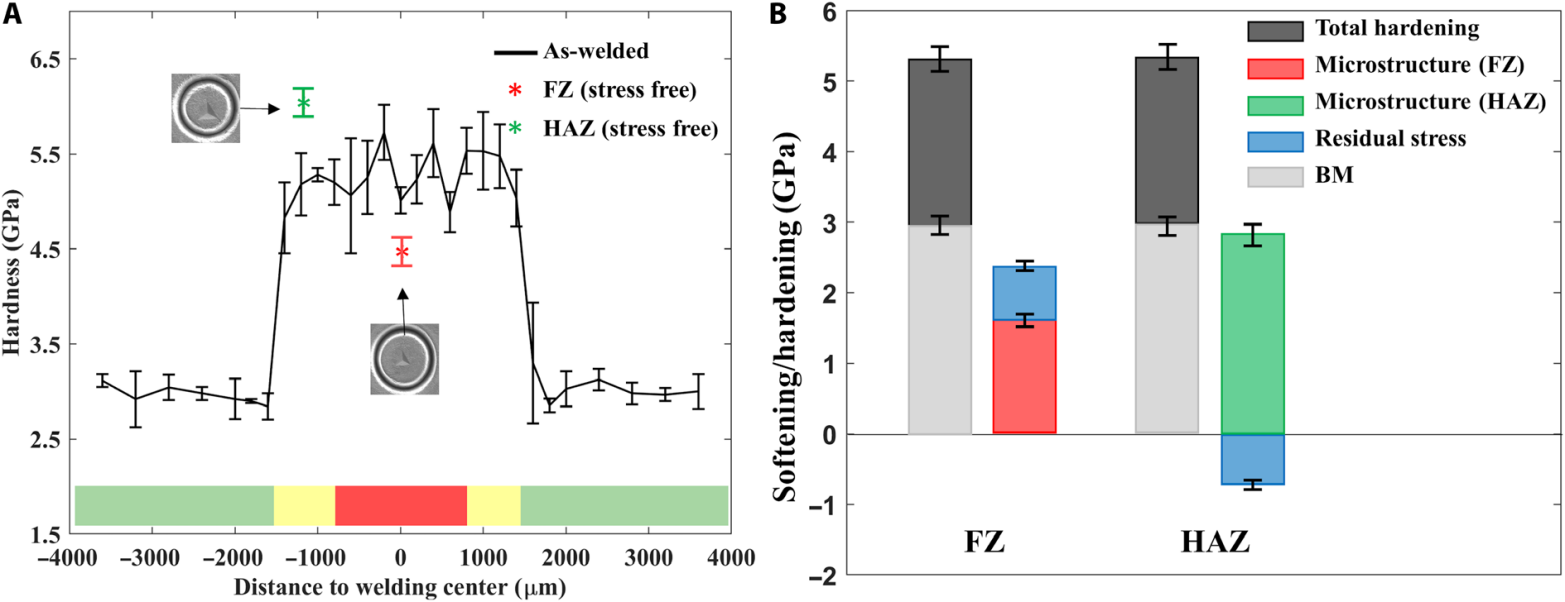
Residual stress measurements using the nanoindentation and residual stress and microstructural effects on microhardness. (**A**) The microhardness distribution across the joint and the average microhardness of the stress-free ring cores in the FZ and HAZ regions. (**B**) Quantitative analysis of residual stress and microstructural hardening/softening in the FZ and HAZ regions.

Local hardness values are dependent on the microstructure (e.g., grain size, the presence of martensite, and precipitate size) and residual stress. The use of nanoindentation measurements enables the quantitative evaluation of the microstructural and residual stress effects on the hardness. The stress-free ring cores provide the hardness value without the residual stress effect, where the hardness depends only on the microstructure. The measured hardness of the weldment (FZ and HAZ regions) lies in the range of 5.1 to 5.6 GPa, where the average magnitude is 5.25 ± 0.31 GPa, while this value drops to 3 ± 0.1 GPa at the HAZ-BM interface (Fig. 5A). The averaged hardness of stress-free ring cores in the FZ and HAZ regions are shown as a dotted line and dash-dot lines, respectively, which indicates that the joined material was microstructurally hardened to 4.51 ± 0.28 GPa in the FZ region and 5.85 ± 0.33 GPa in the HAZ region by grain refinement and the presence of martensite. Looking at Fig. 5B, although the total hardening, i.e., the sum of microstructural and residual stress effects, is the same between the FZ and HAZ regions, the origin of the hardening effect differs. The quantitative microstructural hardening is extracted by subtraction of the hardness of the BM from the hardness of stress-free ring cores, which are 1.51 ± 0.18 and 2.85 ± 0.23 GPa in the FZ and HAZ regions, respectively. Compared with the hardness of stress-free ring cores, the quantitative residual stress effect on hardness is derived from the measured hardness, which is 0.74 ± 0.03 and −0.6 ± 0.02 GPa in the FZ and HAZ regions, respectively.

## DISCUSSION

The results demonstrate the evaluation of multiscale residual stress distribution in the Eurofer97 weldment using Xe^+^ PFIB-DIC ring-core methods. The residual stress distribution in the *x* and *y* directions across the weldment is derived from the master curve fitting by the Xe^+^ PFIB-DIC ring-core method. It is worth reiterating that the asymmetric residual stress profile with sharp peaks at the interface of FZ-HAZ and HAZ-BM was observed in Fig. 2A because of the significant contribution of the nonequilibrium microscale residual stress to the macroscale residual stress distribution in the weldment. The microscale residual stress is related to banded microstructures, texture on the surface, and regions with different microstructures. It is usually self-equilibrated over a length of around three times the grain size when there is no texture effect (*34*, *35*). However, the microscale residual stress is also texture dependent, which means it might become non–self-equilibrium even in a relatively large region if texture exists (*36*). Microscale residual stress can have sufficient magnitude to induce localized tensile residual stress in a region subject to compression on the macroscopic scale (*37*). During the laser welding process, the local heat flow direction usually induces the texture in the weldment (*38*). The thermal gradient and associated phase transformations and solidification result in the severe microstructural misfit at the interface of FZ-HAZ and HAZ-BM. The texture in the FZ region and the microstructural misfits at the interface lead to heterogeneous mechanical properties at the microstructural level, leading to the mismatch between grains and microscale residual stress (*26*). Although conventional residual stress measurements by laboratory-based and synchrotron-based XRD can measure microscale residual stress, it is hard to measure the residual stress precisely in material with strong texture. Such nondestructive residual stress measurements also require a reference sample to calculate the residual stress, which gives rise to unsystematic uncertainties (*12*, *15*). Some destructive techniques such as hole drilling or slitting methods can identify the residual stress at the macroscale. However, because of the limitation of the resolution, the nonequilibrium microscale residual stress is neglected by these techniques (*11*, *13*). Given the high material removal rate of Xe^+^ PFIB and HR analysis of DIC, a proper length scale and resolution can be selected for the ring core, allowing evaluation of macroscale and microscale residual stress simultaneously and study of the microstructural effects on residual stress distribution in high-texture materials. In this project, some of distinct localized microscale strain relaxation was observed around the grain boundaries (Fig. 4, A and C). This is due to the tendency of dislocations to accumulate at grain boundaries, which induces significant microscale residual stress (*18*). Although the texture in the FZ region is slightly higher (just below 4 MRD in the *y* direction) than that in the low-texture HAZ, the texture effect on residual stress is remarkable in the tens-of-micrometer length scale. As illustrated in Fig. 4B, it is assumed that the grain in similar orientation releases the microscale residual strain in the same direction. In this case, the microscale residual strain is not self-equilibrated in the ring-core region, which consequential affects the residual stress distribution in the high-texture FZ region. In contrast, the microresidual stress is self-equilibrated for the ring-core region in the low-texture region, as shown schematically in Fig. 4D. Further evidence is found in the low-texture BM region where conspicuous microscale residual stress is observed, but the macroscale strain relaxation is not evident (Fig. 3, E and F).

Here, the residual stress distribution was also measured using a nanoindentation method by averaging over five line scans. The nanoindentation residual stress measurement is performed with the assumption of equi-biaxial residual stress distribution in the weldments. As shown in Fig. 2D, the equi-biaxial residual stress ($\sigma_{\text{equi}}^{\text{indent}}$) presents the generally symmetric “M-shape” residual stress distribution, which is consistent with the neutron diffraction residual stress measurement on the laser-welded Grade 91 steel (*7*). To directly compare with the PFIB-DIC ring-core method, the equi-biaxial residual stress must be transferred to the non–equi-biaxial. Greco *et al.* (*39*) proposed a methodology to measure the non–equi-biaxial residual stress using nanoindentation. However, this methodology requires the modification of the Berkovich nanoindenter and multiple indents for evaluating non–equi-biaxial residual stress. Lee and Kwon (*40*) proposed a stress ratio (*k*) to extract the two principal residual stress components in an arbitrary non–equi-biaxial state from the average equi-biaxial residual stress. Until now, only a constant stress ratio of 0.33 could be applied to achieve the non–equi-biaxial residual stress evaluation on the weldment (*41*). Use of the Xe^+^ PFIB-DIC ring-core method provides the location-dependent stress ratio (*k*) to transfer the equi-biaxial residual stress to non–equi-biaxial to overcome the primary limitation of nanoindentation residual stress measurement and cross-validate the two techniques. The two principal residual stress components from nanoindentation measurements ($\sigma_{1}^{\text{indent}}$ and $\sigma_{2}^{\text{indent}}$) in Fig. 2D show a similar trend to that of the ring-core method (Fig. 2C). However, some unexpected inconsistency occurs at the position where the nonequilibrium microscale residual stress exists, e.g., high-texture FZ region and microstructural transition areas. This reveals the limitation of nanoindentation residual stress measurement, with its intrinsic difficulty of selecting a truly stress-free reference and stress ratio for non–equi-biaxial residual stress calculation in complex material systems. On the one hand, the sharp microstructure and residual stress gradients usually exist in these transition regions, which implies that the location-dependent stress-free reference at HR is desirable to improve the quality of the nanoindentation residual stress measurements. On the other hand, the uniform stress ratio in the high-texture region is difficult in providing the accurate stress ratio for non–equi-biaxial residual stress evaluation. Further work is needed to optimize the location-dependent stress-free reference and macroscale stress ratio at the narrow transition areas using PFIB-DIC technique to develop the next generation of advanced nanoindentation residual stress measurement, leading to more accurate measurement with reduced uncertainty.

In addition, combining the two techniques enables the quantitative analysis of the microstructural and macroscale residual stress hardening (Fig. 5, A and B), enabling evaluation of the underpinning mechanisms of degradation of mechanical property of the laser-welded weldment. Microstructural hardening is the result of the presence of precipitates, grain boundary strengthening, and the martensite transformation in the FZ and HAZ regions (*42*–*44*). The compressive residual stress aggravates the hardening phenomenon in the FZ region, while the tensile residual stress softens the material in the HAZ. Both methods enabled consideration of the macroscale biaxial residual stress, achieving an improved understanding of residual stress distribution in the weldment. As the Xe^+^ PFIB-DIC technique does not require a reference, this technique provides a more reliable residual stress distribution in the transition areas.

The Xe^+^ PFIB-DIC technique can also map time-resolved HR microscale strain relaxation, which provides insight into analyzing the void formation and creep cracking caused by residual stress relief. It has been widely reported that the creep cracks are a crucial challenge for ferritic-martensitic steels under load at high temperature, and residual stress is often considered as the most primary reason for microcracking (*45*, *46*). The high operating temperature triggers the thermal relaxation of welding-induced residual stress. The accumulation of microscale strain relaxation during the thermal residual stress relief leads to the formation of the voids and creep crack initiation (*47*–*49*). Such accumulation during the residual stress relaxation can be visualized by the time-resolved HR strain relaxation maps. It can be seen in Fig. 3 that even in the region that is subject to compressive macroscale strain, the microscale residual strain is sufficient to form tensile localized residual strain. The use of time-resolved HR strain maps is valuable for identifying and quantifying sharp microscale strain relaxation gradients and strain accumulation during residual stress relief. It provides critical information necessary for developing a micromechanical rationale for failure. The Xe^+^ PFIB-DIC technique, however, has some limitations that require careful consideration. Because of the SEM’s serial acquisition, the pixels in the *x*, *xy*, and *y* directions are in order of increasing scanning time gap (*20*); this results in the broadening of the range of the 95% confidence interval in the *xy* and *y* directions compared to the *x* direction. In addition, although the type I, II, and III strain relaxations are identified on the basis of their locations, further work is still necessary to quantitatively characterize the magnitude of type I, II, and III residual stresses on the time-resolved HR strain relaxation maps.

In conclusion, our analysis of the heterogeneous microstructural and residual stress distribution in laser-welded Eufofer97 steel, through the combination of EBSD, Xe^+^ PFIB-DIC, and nanoindentation, offers new tools for evaluating multiscale residual stress and providing insight into the critical engineering challenges of laser-welded Eurofer97 steel and other complex weldments. With the Xe^+^ PFIB-DIC ring-core method, the macroscale residual stress is characterized across the weldment, and the microscale residual stress is observed from time-resolved HR strain relaxation maps. The nonequilibrium microscale residual stress in high-texture regions affects the macroscale residual stress, rationalizing the asymmetric residual stress distribution. The time-resolved HR strain relaxation map was used to visualize the microscale residual strain field—the precursor of void formation and crack initiation during residual stress relief at elevated temperatures. The mechanistic connection between residual stress, microstructure, and microhardness is established, where the residual stress contributes around 25% to hardening and softening in the FZ and HAZ, respectively. Further analysis enables extraction of a coefficient using the Xe^+^ PFIB-DIC technique. This method enables the reconstruction of the residual stress from equi-biaxial to non–equi-biaxial by considering texture effects. This has great potential to overcome the current limitation of nanoindentation residual stress measurement. The Xe^+^ PFIB-DIC ring-core method is now available for the evaluation of HR multiscale residual stress heterogeneity in welded in-vessel fusion components and even more complex material system.

## MATERIALS AND METHODS

### Materials

The as-received 6-mm-thick Eurofer97 steel (made by Böhler Austria GmbH) had a composition Fe-0.11C-8.82Cr-1.08W-0.13Ta-0.48Mn-0.2V (in weight %). Rieth *et al.* (*43*) have described the detailed fabrication and heat treatment of as-received Eurofer97 steel in a previous study. The Eurofer97 plate butt-weld was made from two plates, each 150 × 75 × 6 mm^3^. The single-pass laser weld was made using a Yb-fiber laser source with a spot size of 200 μm and a welding speed of 1.2 m/min at The Welding Institute (TWI) to attain a high-quality weld (narrow penetrating bead and slightly concave). As shown in Fig. 1A, the as-welded sample used in this research was cut from the as-welded Eurofer97 plate by electrical discharge machining, and the size was ~25 × 6 × 6 mm^3^.

The microstructure was characterized using a Jeol-7100F SEM, equipped with a Thermo Fisher Lumis EBSD detector. Suitable sample preparation processes were applied in a sequence of mechanical polishing, vibration polishing using 0.3-μm colloidal silica, and etching with Vilella’s reagent (1 g of picric, 5 ml of HCl, and 100 ml of ethanol). The 19 EBSD maps were captured with a resolution of 512 × 384 pixels using an accelerating voltage of 20 kV and beam current of 12 nA with a step size of 0.6 μm and a 2 × 2 pattern binning. Pathfinder software was used to collect the EBSD orientation data, and MTEX 5.2.8 software was applied to analyze the crystallographic orientation, calculate the texture intensity, and average Young’s modulus and Poisson’s coefficient. The postprocessing of the raw EBSD orientation data began with filtering, which filled the missing data using the interpolation method of the nearest neighbor. Denoising was then performed according to the deviations from the true orientation. The grains were reconstructed by reducing the orientation noise with a lower threshold of 15°. The grain morphology in the region of 4750 × 200 μm^2^ (dashed rectangle in Fig. 1A), which covers FZ, HAZ, and BM, was visualized by stitching the 19 separate EBSD maps. The IPFs characterize the texture intensity of each EBSD map in both the *x* and *y* directions. The highest texture intensity in the *x* and *y* directions that is extracted from each EBSD map is used to describe the texture in the corresponding area. An example of IPFs from one of the EBSD maps is the inset in Fig. 2B, which describes the texture intensity at the area around −200 μm to the welding center. The texture observed in this area is 2.2 MRD parallel to {011} in the *x* direction and 4 MRD parallel to $\left\{\bar{1}11\right\}$ in the *y* direction. The average Young’s modulus and Poisson’s coefficient in the *x* and *y* directions for the ring-core regions in the FZ and HAZ were derived according to their crystallographic orientation using MTEX 5.28 in MATLAB and applied to study the anisotropic residual stress in the *x* and *y* directions (*50*–*52*). The as-welded Eurofer97 is assumed to exhibit cubic crystal symmetry. The ring core in the BM region is consider as isotropic, where a Young’s modulus and Poisson’s coefficient from the parent materials is assumed. The elastic constants of Eurofer97 have been studied previously (*53*).

### Xe^+^ PFIB-DIC ring-core method

The Xe^+^ PFIB-DIC ring-core method comprises two procedures: SEM image acquisition during incremental Xe^+^ PFIB milling (Fig. 1C) and residual stress measurement (Fig. 1, D and E). The first procedure was performed using a TESCAN MIRA 3 PFIB-SEM. Given that the reliable bulk behavior can be obtained if at least eight grains are contained in thickness direction (*54*), the inner diameter of the ring core (*d*) of 30 μm at the FZ region and 20 μm at the HAZ and BM regions enabled sufficient grains to be captured at different regions within a reasonable data acquisition time. The final milling depth (*h*) was equal to the inner diameter, and a uniform milling depth was used for each incremental step. Fifty milling steps were performed at a beam energy of 30 keV and beam current of 15 nA, which achieved full relief of residual stress (i.e., when *h/d* = 1). Compared to the Ga^+^ FIB, the primary advantage of the Xe^+^ PFIB is the achievement of much faster material removal without any amorphization to materials during the milling process (*22*). This advantage ensures that the residual stress is not induced during the material removal procedures. Ten secondary electron (SE) images were acquired with a spot size of 10 nm in every incremental step to record the strain relaxation. The processes of incremental milling and SE image acquisition were performed with the help of a specifically developed automated program by TESCAN s.r.o.

Customized MATLAB-based DIC software was used to perform strain relaxation correlation (*55*). The first step of DIC was the correlation analysis over the stack of SE images. A Gaussian filter was used to denoise the raw SEM images, and a fiducial mesh was used to track the ring-core deformation of the central region to avoid the stress concentration at the pillar edge. The fiducial mesh in the space of 20 pixels was used to record ring-core displacements to achieve a high spatial resolution of 200 × 200 nm^2^ (Fig. 1D). The pre-set “subset” (31 × 31 pixels) was used for tracking a single node movement, which ensured that single node movement occurred only in the subset region with a minimum of boundary effects (*56*). The outline shape of the markers is highlighted in Fig. 4 (A and C), representing the area measured by the DIC in the ring core. The correlation results for 10 SE images in the same incremental milling step were averaged to reduce noise. The outliers (poorly tracked nodes) were removed in postprocessing to estimate ring-core displacements in high accuracy and precision. The detail of the outlier removal process has been reported previously (*31*). The HR ring-core displacements were analyzed by tracking the node movements, as shown in Fig. 4 (A and C). The gradient of the single node movement was evaluated to quantify the ring-core strain relaxation. To visualize time-resolved HR strain relaxation maps, the strain values of each node were converted into a color-coded map for each incremental milling step.

Last, the linear fit to all node movements, with their fiducial position, yielded time-resolved strain relaxation for each incremental milling step using the least-squares function. The time-resolved strain relaxation was fitted to the master curve function to obtain the full in-plane strain relaxation components accompanied by the error bars that correspond to a 95% confidence interval (CI) (*30*). The residual stress was then calculated by generalized Hooke’s law, where the out-of-plane stress is negligible (*27*)

$$\sigma_{x}^{\text{PFIB}}=-\frac{E_{\text{avg}}^{x}}{\left(1-v_{\text{avg}}^{x}v_{\text{avg}}^{y}\right)}[\Delta \varepsilon_{\infty }^{x}+v_{\text{avg}}^{x}\Delta \varepsilon_{\infty }^{y}]$$ (1)

$$\sigma_{y}^{\text{PFIB}}=-\frac{E_{\text{avg}}^{y}}{\left(1-v_{\text{avg}}^{x}v_{\text{avg}}^{y}\right)}[\Delta \varepsilon_{\infty }^{y}+v_{\text{avg}}^{y}\Delta \varepsilon_{\infty }^{x}]$$ (2)

where $\Delta \varepsilon_{\infty }^{x}$ and $\Delta \varepsilon_{\infty }^{y}$ are the strain relaxations, and σ*_x_* and σ*_y_* are two in-plane residual stress components in the *x* and *y* directions. $E_{\text{avg}}^{x}$, $E_{\text{avg}}^{y}$, $v_{\text{avg}}^{x},$ and $v_{\text{avg}}^{y}$ are average Young’s modulus and Poisson’s coefficients computed according to the crystal orientation in the ring-core area in the *x* and *y* directions, respectively.

### Nanoindentation

The nanoindentation measurement was carried out using an Agilent G200 nanoindenter with a Berkovich indenter tip at the Materials Research Facility, United Kingdom Atomic Energy Authority (UKAEA). Depth-controlled indentations were carried out to 1.5-μm depth using continuous stiffness mode (2-nm amplitude, 45-Hz frequency, 0.05 μm/s), and the mechanical properties were determined according to the theory by Oliver and Pharr (*57*). An array of 5 × 40 indentations was performed on the as-welded sample (see Fig. 1A). The nanoindentation measurements in the *x* direction were in the space of 200 μm, while the interval between nanoindentation in a column in the *y* direction was 30 μm. Because the ring core is considered notionally stress free, the location-dependent stress-free nanoindentation tests were carried out on the ring cores at the FZ, HAZ, and BM regions. The setup for executing a stress-free nanoindentation test was the same as performing a nanoindentation array scan. To avoid error because of insufficient stiffness of the reference ring core, the hardness values reported are an average of the depth-resolved hardness from 100 to 500 nm deep, and the load and contact area were extracted for a depth of 500 nm for residual stress evaluation. The residual stress from nanoindentation measurement ($\sigma_{\text{equi}}^{\text{indent}}$) is assumed to be equi-biaxial and uniform in the near-surface region in this technique (*58*, *59*). The method of deriving the residual stress from comparing the difference regarding load-depth curves and contact areas between residual stress and stress-free states has been discussed elsewhere (*33*, *59*). To cross-validate the results from the PFIB-DIC technique, the equi-biaxial residual stress ($\sigma_{\text{equi}}^{\text{indent}}$) was transformed to the non–equi-biaxial residual stress (major and minor principal stress $\sigma_{1}^{\text{indent}}$ and $\sigma_{2}^{\text{indent}}$) via the location-dependent stress ratio *k* at corresponding position in the following manner

$$k=\frac{\sigma_{1}^{\text{PFIB}}}{\sigma_{2}^{\text{PFIB}}}\;(-1<k<1\;\text{and}\;k\neq 0)$$ (3)

$$\sigma_{1}^{\text{indent}}=\frac{3(L_{0}-L)}{(1+k)A_{C}}$$ (4)

$$\sigma_{2}^{\text{indent}}=k\sigma_{1}^{\text{indent}}=\frac{3k(L_{0}-L)}{(1+k)A_{C}}$$ (5)

where the $\sigma_{1}^{\text{PFIB}}$ and $\sigma_{2}^{\text{PFIB}}$ are the major and minor principal residual stress components from the PFIB-DIC ring-core method, respectively.
